# Art therapy/occupational and play therapy: plastic expressiveness as a means of reducing loneliness, anxiety, sadness – research carried out during the period 2008-2022 with the theme: creation in / with elements from nature at the placement center

**DOI:** 10.1192/j.eurpsy.2024.1091

**Published:** 2024-08-27

**Authors:** E. Chirila

**Affiliations:** Centru de zi de recuperare pentru copii cu autism. Centru de servicii de recuperare neuromotorie și de tip ambulatoriu pentru persoanele adulte, Direcţia Generală De Asistenţă Socială Şi Protecţia Copilului Cluj, Cluj-Napoca , Romania

## Abstract

**Introduction:**

*Occupational therapy - which also includes art therapy - is an activity/test with a purpose, it involves coordination between the sensory, motor, cognitive, and psychosocial systems of the individual. “Sciences recognize the role of observation in research… All artists who practice art therapy are based on their own artistic activity and present a common recurring feature: they are always in line with “essential pragmatism”.* (McNIFF, Shaun, Trust the process: an artist’s guide to letting go. Creative ability. Psychological aspects. Self-actualization (Psychology). Artist-Psychology, Shambhala Publication, Boston, 1998, p. 78)

**Objectives:**

We seek to find new development solutions through stimulation, creativity, catharsis, and socialization to be authentic, spontaneous, feel fulfilled, emotionally balanced, and transformed, with the aim of fulfilling one’s social role through contact with human and environmental factors. (Emilia Chirilă, ART THERA PY IN EMOTIONAL DISORDERS OF CHILDREN AND ADOLESCENTS, printed edition 2018) ISBN 978-973-0-27683-1)

**Methods:**

Through the graphic gesture, the child expresses various issues related to his feelings, like the search for his identity, the generated anxieties, the family and professional environment, and the situations of neglect and abuse. (MALCHIODI, Cathy A, Handbook of Art Therapy, The Guilford Press, New York and London, 2003, p. 157).

**Results:**

The following reactions can be identified: aggression, frustration, dominance tendency, low self-esteem, fraternal rivalry, hopelessness, sadness, compensation mechanisms, self-defense, other significant psycho-traumatic aspects. The disappearance of frustrations and negative feelings due to the disinterest of parents who do not visit the beneficiaries was achieved by gaining authority over the environment and by improving pre-existing skills. emotional disorders of children and adolescents” - Journal of Neurology and Psychiatry of Children and Adolescents from Romania - 2012 - vol. 15 - no. 3- p 121-136 - ISSN (printed): 2068-8040)

**Image:**

**Figure:**
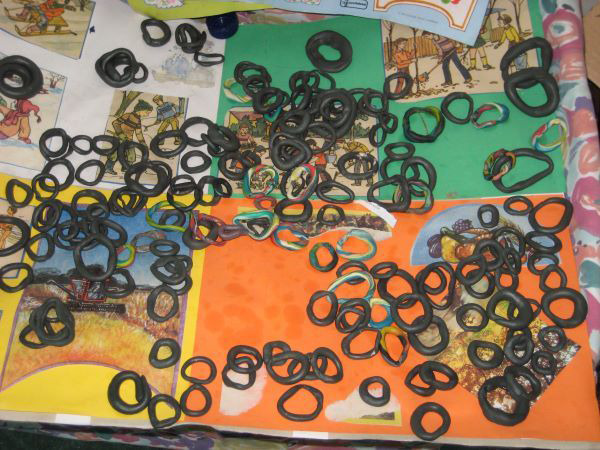

**Image 2:**

**Figure:**
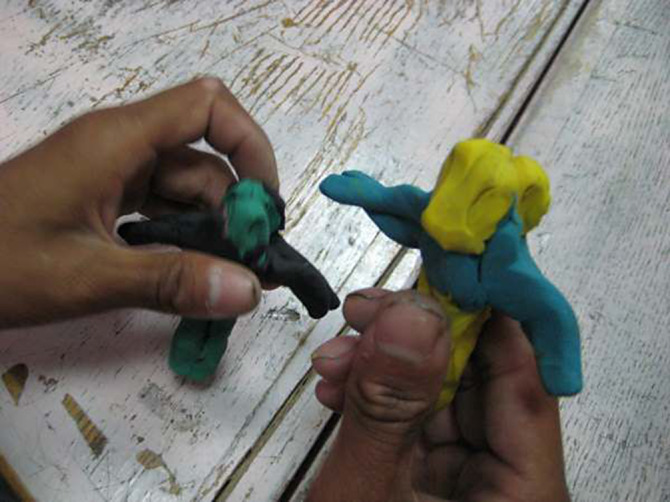

**Image 3:**

**Figure:**
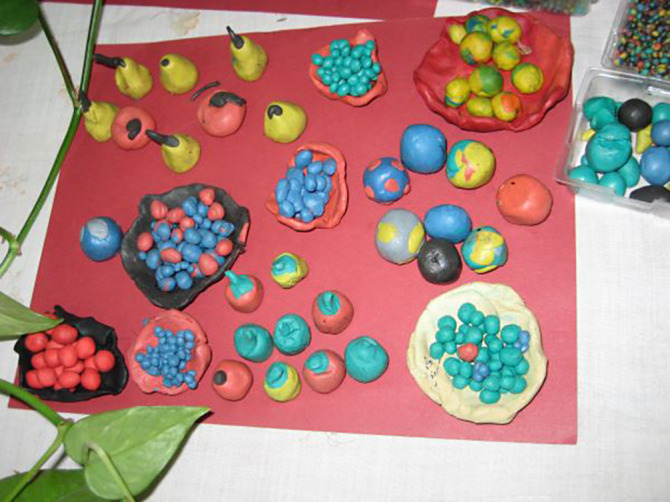

**Conclusions:**

Through the creative process and symbolic communication, associated with narration and imitation, we realize the development of outstanding and hidden abilities, we develop new ways of communication, new ways of self-expression, and new ways of seeing things, to increase the ability to face existential problems.(2018 Emilia Chirilă - Art therapy in emotional disorders of children and adolescents: “Festina lente - Hurry slowly!” Harmonizing the rhythm with those around us ,print edition, ISBN 978-973-0-27683)

**Disclosure of Interest:**

None Declared

